# Supplementary material for: Are carbon emissions trading and green financial instruments synergistic? -Comprehensive quantitative research based on content analysis
Source: PLoS One. 2024 Mar 7;19(3):e0298601. doi: 10.1371/journal.pone.0298601 (PMC10919601; doi:10.1371/journal.pone.0298601)
Supplement: S1 Data — (ZIP) [file pone.0298601.s001.zip › Data/地级市Z_F工作报告.zip!/╣π╢1⁄2/╜¡├┼╩╨/└┤╫╘╣1⁄2╓┌║┼í╛┬φ┐╦╩2╛▌═°í┐_2007.docx]

各位代表：

　　我代表市第十二届人民政府向大会作政府工作报告，请予审议，并请市政协各位委员和其他列席人员提出意见。

工作回顾

　　本届政府任期的２００３年至２００６年，是我市全面落实科学发展观、改革开放和现代化建设取得新的重大成绩的时期。在省委、省政府和市委的正确领导下，在市人大及其常委会和市政协的监督支持下，市政府高举邓小平理论和“三个代表”重要思想伟大旗帜，坚持以科学发展观统领经济社会发展全局，扎实推进社会主义和谐社会建设，大力实施工业强市、民资外资富市、科教兴市、环境优市战略，抢抓机遇，开拓进取，有效化解经济社会发展中的突出矛盾和问题，战胜百年一遇特大洪灾、强等自然灾害，较好地完成了市第十二届人民代表大会和“十五”计划确定的各项目标任务，为时期经济社会又好又快发展奠定了坚实基础。

　　——经济发展步入快车道，综合实力显著增强。全市ＧＤＰ由２００２年的５６６亿元增加到２００６年的９２１亿元，增长速度逐年加快，由８．５％提高到１５．３％，创近１１年新高，年均增长１２．８％。三次产业比例由１３：４７：４０发展为９：５２：３９。２００６年地方财政一般预算收入达５０．６６亿元，比上年增长２１．６８％，连续四年超过全省财政收入平均增幅，比２００２年翻了一番，年均增长１８．９２％，占ＧＤＰ的比重由４．５％提高到５．５％。社会消费品零售总额由２１９．８９亿元增加到３５２．３４亿元，年均增长１２．５％。城乡居民储蓄存款余额由５７８．３５亿元增加到９４１．０３亿元，年均增长１２．９４％。

　　——镇级经济实现新突破建设扎实推进。强镇富民成效显著，２００６年镇级地方财政一般预算收入２１．２５亿元，比２００２年翻了一番，占全市财政一般预算收入的４１．９４％。２００３年以来，新增财政收入超亿元、超千万元的镇（街）分别有３个和１６个。８个镇（街）荣获全国千强镇称号。对″三农″扶持力度加大，各级财政投入达３２亿元，年均增长１８．６％。现代农业加快发展，全市农业产业化组织达２６３个，其中农民专业合作经济组织１５９个，龙头企业１０４家，带动农户２６万多户。农作物和畜禽良种覆盖率分别超９５％和９３％。建立３２家农业科技创新中心、示范基地，新增９个国家级绿色食品和６个省级名牌产品。农村生产生活条件明显改善，２００６年农村居民人均纯收入５５９２元，比２００２年增长２６．２％。实现了１９９８年市人大议案提出的水利安全达标加固工程目标。农村自来水和液化气普及率分别达８１％和８９％，３７０条村２０．７万人的饮水难问题基本解决。农村公路硬底化建设惠及群众６０多万人。完成农村危房改造３１７４户。３１２条年集体收入３万元以下的贫困村实现脱贫。新型农村合作医疗加快推进，覆盖率达７８．１５％，比２００２年提高５１．３５个百分点。农村综合改革逐步深化，取消农业税，农民人均税费减负率达９９．９％。全市各级财政投入９０００多万元全面实施农村免费义务教育，３４万多名农村学生受益。

　　——自主创新迈出重大步伐，产业结构调整成效突出。产学研联合基地和省市级工程中心分别由２００２年的３５家和２７家，发展到１１５家和５５家。成立市科技创业中心。省级技术创新专业镇达１４个，居全省第二。产业公共服务平台建设初见成效，省级摩托车检测中心挂牌成立，引进广天机电工业研究院和华南精细化工研究院。国家信息化试点城市建设扎实推进，信息化综合指数达７４％，比２００２年提高２３．４７个百分点。品牌带动战略成效显著，新增８个中国名牌产品、１０个中国免检产品、２个中国驰名商标和４９个省名牌产品、４４个省著名商标。

　　产业发展取得新突破。２００６年全市规模以上工业增加值达４１０．６亿元，比上年增长２１．７６％，比２００２年增长７７．６２％。新增９个国家级产业基地，创建４个省产业集群升级示范区，形成了纺织化纤、摩托车、不锈钢等一批超百亿元的产业集群。电子信息、汽车零配件、精细化工等新兴产业发展迅速。市高新区、江沙工业走廊和银洲湖区域规模以上工业总产值分别达到或接近１００亿、２００亿和３００亿元。产业适度重型化和高级化趋势增强，重工业比重由２００２年的３３．９％提高到４３．４％。高新技术企业由２００２年的８４家增加到１８０家。高新技术产品产值由２２０亿元增加到６３０亿元，增长近２倍，占工业总产值比重由１５．６％提高到２８％。第三产业增加值由２００２年的２２８．５５亿元增加到３５８亿元，年均增长１０．９７％。物流业方兴未艾，成功举办首届物流合作推介会，签约金额达７１．７亿元。初步打响了摩托车博览会、新会五金不锈钢产品展览会、水口水暖卫浴展销洽谈会、开平牛仔服装节、恩平麦克风及电声视频产品国际展销会等会展品牌。旅游业发展加快，新建了逸豪、丽宫国际等五星级标准酒店，锦江温泉、古兜温泉被评为国家４Ａ级景区，恩平市被命名为全国首个“中国温泉之乡”和“地热国家地质公园”，新开发恩平歇马村、崖门古战场等一批旅游景区。２００６年接待游客６０７万人次，旅游总收入５２．５亿元。

　　——民营经济不断壮大，外源型经济快速发展。２００６年全市民营企业户数和注册资金分别达１．５９万户、１９３．３０亿元，比２００２年增长４６．１８％和１．６３倍。规模以上民营企业实现工业总产值５９８亿元，占全市工业总产值比重由２００２年的２７．６％提高到３４．５％。对外贸易迈上新台阶，进出口总额突破１００亿美元，其中出口７４．２９亿美元，比２００２年增长１．５倍，年均增长２６％。２００３年以来累计实际利用外商直接投资２６．４６亿美元，超千万美元的外资项目１６４个；来自欧美、日韩等发达国家和地区的投资由２００２年的１．１１亿美元增加到２００６年的２．０７亿美元，占全市实际利用外商直接投资比重由１５．１％提高到３０．５３％。成功举办世界银行中国投资环境论坛和粤台经贸交流会，连续三年与香港贸易发展局分别在欧美日联合举办“香港／江门推介会”。

　　——基础设施建设实现大跨越，城乡面貌呈现新气象。２００６年全社会固定资产投资２７９．２５亿元，比上年增长１７．６％，２００３年以来累计完成８７８亿元。投资结构进一步优化，基础设施和基础产业建设强力推进。开阳、江鹤二期和中江高速公路建成运行，江珠高速、滨江大道即将通车，广珠城际轨道交通工程江门支线建设进展顺利。全市高速公路通车总里程达３３０公里，比２００２年增加９５公里；公路通车总里程达６８２８公里，比２００２年增加５２８公里，实现了市到各县级市通高速公路、县到镇通二级以上公路、镇到行政村通四级以上公路的目标。完成了３２５国道开平、恩平段路面改造，全省最长的公路隧道彩虹岭隧道全线贯通。建成江门、新会和鹤山新汽车客运站，７９．３％的行政村通班车。新会区实现与蓬江区并网供水。广海湾及川岛深水港开发方案研究成果通过专家评审。崖门５０００吨级出海航道整治主体工程完成，劳龙虎水道整治工程进展顺利。台山电厂一期３００万千瓦、新会双水发电厂二期３０万千瓦工程建成投产。台山直购电试点成功启动，成为全国第一个“点对多点”直购电试点地区。编制并实施新一轮城市总体规划，城镇化水平由２００２年的３８．８％提高到５６％。撤并镇村、镇改街、村改居进程加快，建制镇由９２个撤并为６２个。

　　——节约型经济加快发展，环境保护取得新成绩。我市被列为省建设节约型社会试点城市。４家企业列入第一批国家和省循环经济试点单位，银洲湖纸业基地成为省循环经济试点园区，台山再生资源工业集中区获得国家环保总局批准。荣获“国家卫生城市”、“国家环境保护模范城市”称号和“中国人居环境奖”。每新增亿元ＧＤＰ新增建设用地由２００２年的３４公顷减少到９公顷，下降７３．５％。主要污染物化学需氧量排放总量比２００２年下降４７％。新建成６家城镇污水处理厂，完成江门河综合整治工程，会城河、紫水河综合整治取得成效。台山电厂一期机组和新会双水发电厂脱硫工程达到国内先进水平。水泥企业消烟除尘整治成效显著，工业危险废物和医疗废物管理进一步规范。市区生活垃圾无害化处理率由２００２年的３８．３９％提高到６４％，生活污水处理率由３０％提高到６５％。建成区绿化覆盖率和人均公共绿地分别由３８．１９％和８．８３平方米提高到４０．３０％和９．３５平方米，环境空气质量日报优良率保持在９５％以上。鹤山市、新会区、开平市成功创建省林业生态县。

　　——体制改革取得重大进展，经济发展活力增强。企业改革深入推进。全市实施改制企业１０６４户，改制面达９０％，安置职工８万人。其中市政府授权经营的２１２户国有企业改制面由２００２年初的１５．１％扩大到９６．３％，安置职工４．３万人。建立健全三级国有资产管理体制，市直国有企业总体实现扭亏为盈。完成了江门甘化股份公司、开平春晖股份公司和新会美达股份公司的股权分置改革，江门益胜浮法玻璃公司中方股权及债权成功实施整体转让，恩平广联泰、开平涤纶集团和江门造纸厂依法实施破产，江门中旅集团与有关金融机构签订了债务重组协议，金羚电器公司成功引入战略合作伙伴。机构改革、行政审批制度改革和公共财政体制改革取得重要进展，全面完成事业单位人事制度改革，投资体制改革稳步推进，农村信用社体制改革试点工作取得阶段性成果。

　　——精神文明建设取得新成效，社会事业全面进步。成功创建省文明城市，群众性精神文明创建活动蓬勃开展。文化名市建设初见成效，开平与村落成为中国申报２００７年世界文化遗产项目，顺利通过联合国专家评估；台山广东音乐列入首批“国家级非物质文化遗产”；崖门海战历史文化和梁启超、陈垣等名人文化得到有效保护。建成五邑华侨华人博物馆、江门美术馆、江南文化广场、新会名人广场、市老干活动中心等一批文化馆场以及院士路、恩平荣誉市民大道等，成功举办华人嘉年华、侨乡旅游节、中国曲艺节、书香节等一系列活动，东湖广场被评为省“十佳文化广场”和“全国特色文化广场”。组织了文化服务百镇千村行系列活动。教育事业有新发展，组建江门职业技术学院并建成新校区，创建４个省教育强镇（街）、７所国家级示范性普通高中和１１所国家级重点中等职业学校，举办了２００６江门职业教育成果展示暨蓝领人才推介交流会。建成江门一中新校区、新会实验中学、恩平实验中学、台山新宁中学和育英学校，完成２８４所中小学布局调整和８１所老区、山区学校危房改造。高中阶段教育和高等教育毛入学率分别达８１．７％和２７．１９％，比２００２年提高８．７和５．８个百分点。全市人才资源总量达３０．６５万人，比２００２年增长２２．４５％。

　　卫生、体育等社会事业取得新成绩。疾病预防控制、医疗救治、卫生监督三大体系进一步健全。建立起疫情信息网络系统，公共卫生突发事件预警和应急机制更加完善。市疾控中心和新会区、鹤山市疾控中心建成使用，防控人禽流感、非典型肺炎、艾滋病等重大传染病工作取得新进展。妇幼保健、精神卫生工作水平提高。全面完成省下达的人口与计划生育任务。慈善公益万人行活动成效显著。建成市体育公园一期、新会体育馆，民办体育场馆大量涌现，群众性体育活动蓬勃开展，“国家网球训练基地”落户我市，五邑运动员在国内外重大赛事中取得优异成绩。老龄工作得到加强，扩大了老年人免费或优惠乘车的范围，并为免费乘车的老年人购买意外保险。残疾人就业康复水平提高。国防动员、民兵预备役和双拥工作进一步加强，荣获“全国双拥模范城”三连冠和“广东省双拥模范城”五连冠。侨务工作取得新突破，海外联谊、对外宣传、维护侨益与服务侨胞等工作有新发展。

　　劳动和社会保障工作成效突出。新闻、出版、人防、档案、方志、宗教、气象等工作有新进步。２００３年以来新增城镇就业岗位逾１０万个，安排城镇劳动力１３万人，其中３．５万多名下岗失业人员实现再就业。城镇登记失业率由２００２年的３．１３％下降到２．６３％。企业职工月最低工资标准从３８０元提高到６００元。２００６年全市城镇在岗职工人均工资收入１６５３０元，比２００２年增长４８％。清理建设领域拖欠工程款和农民工工资工作取得阶段性成果。加强劳动监察，建立解决企业欠薪机制。社保扩面征缴力度加大，参加养老、失业、医疗和工伤保险人数分别达４６万、４０万、４７万和３６万人。人均居住面积６平方米以下的困难户住房问题得到解决。

０３”、“粤鹰”、“打黑除恶”等专项整治行动成效显著，社会治安进一步好转。贯彻落实《信访条例》，建立健全处理信访突出问题及群体性事件联席会议、信访督查员、部门领导公开接访和基层“五长”联合接访等制度，社会矛盾纠纷调解机制进一步健全。２００６年县级以上信访部门受理群众来信来访及越级上访数量均比上年下降近４０％。加强镇村安全监督管理，推进行业安全监管标准化建设，安全生产形势明显好转。开通江门市食品安全信息网，食品药品、消防、道路交通安全以及“三防”等关系群众和社会安全的基础工作得到强化。基层武装建设更加巩固，民兵预备役队伍在处置突发事件、抢险救灾中发挥了重要作用。

　　——民本政府建设上新台阶，为民服务水平显著提高。自觉接受市人大及其常委会的监督，认真执行市人大及其常委会的决议。定期听取人大代表和政协委员的意见建议，并创新听取方式，变“领导点题”为“代表选题”。２００３年以来共办理人大代表议案３件、建议６１４件，办结满意率９７．４％；办理政协提案６１３件，办结满意率９９．４％。认真实施行政许可法，依法行政水平进一步提高。扎实推进电子政务工程，在全省率先开通政府网上行政服务系统，开设网上“侨乡论坛”，推行“群众点题”等政务公开方式，设立“１２３４５”政府为民服务热线，启用行政审批电子监察系统。建立全市经济形势分析联席会议和经济社会发展顾问咨询制度，开发城市经济发展预测与决策支持系统软件，制定并实施《江门市可持续发展战略纲要》。调整和清理行政审批事项，市级审批事项由２００２年的５４４项减为２１０项。在市直机关全面推行ＩＳＯ质量管理体系，已有５１个单位通过认证，有效提高了政府系统的执行力，得到国家和省行政管理学会的充分肯定。实施推诿、搁置追究制，健全投诉机制，政府工作的规范性和效能大幅提高。政府廉政建设和反腐败工作扎实推进，查处了一批违纪违法案件。在世界银行公布的２００６年中国城市投资环境评价中，我市在政府效率方面名列前茅。

　　回顾本届政府的工作，我们深深体会到，江门经济社会要实现全面协调可持续发展，关键是要坚持以科学发展观为统领，切实把中央和省的方针政策与江门实际紧密结合，按照市委的决策部署创造性地开展工作；坚持走新型工业化道路，千方百计提高自主创新能力，加快产业结构转型升级，促进经济增长方式转变；坚持把招商引资作为经济工作的重中之重，发挥侨乡优势，积极引进海内外资金、先进技术和管理经验；坚持把实现好、维护好、发展好最广大人民群众的根本利益作为政府工作的出发点和落脚点，努力解决人民群众最关心、最直接、最现实的利益问题；坚持推进民主法制建设和政府管理创新，营造法制、人才、政务、交通、生活等“五好环境”。

　　各位代表，本届政府取得的成绩，是在省委、省政府和市委的正确领导下，全市人民贯彻落实“三个代表”重要思想和科学发展观，团结奋斗，开拓进取的结果；是各级人大及其常委会和政协监督支持的结果；是中央、省驻我市单位及军警部队、各民主党派、人民团体、社会各界人士、外来投资者和建设者、港澳台同胞、海外侨胞关心支持的结果。在此，我代表市人民政府，向关心支持参与江门经济社会发展建设的市民们、朋友们、同胞们、侨胞们表示崇高的敬意和衷心的感谢！

　　我们也清醒地看到，我市经济社会发展还存在不少突出矛盾和问题。主要是：粗放型经济增长方式还没有根本性转变；产业竞争力、自主创新能力和现代服务业发展水平与先进地区相比仍有较大差距；区域经济发展不平衡问题依然突出，农民收入增长缓慢；企业转制、土地征用、城镇拆迁、环境保护等问题引发的社会矛盾增加；教育、卫生、社会保障、城市管理、社会治安、政府建设等方面还存在薄弱环节。对这些问题，建议新一届政府采取更加有效的措施加以解决。

今后五年的奋斗目标和主要任务

　　今后五年，是我市在科学发展轨道上加快发展的重要时期。总体发展环境有利，世界经济稳中趋好，国际产业转移向中高端升级；全国、全省保持良好发展势头。我市经济社会发展进入加速阶段，区位、基础设施、资源和环境优势日渐凸显，全市广大干部群众及海外乡亲共谋发展、齐心思进的氛围浓厚，我市后发争先的主客观条件已基本具备，但我们也面临珠三角各市产业升级步伐加快，省内东西两翼和山区市超常规发展的新局面、新挑战。江门作为珠三角一员，必须在全省全面建设小康社会、率先基本实现社会主义现代化进程中发挥更大作用。对此，市委站在全局和战略高度，对我市未来五年的发展作了全面部署，市第十一次党代会明确提出“五大发展战略”及“两滨驱动”、“一带联动”的发展谋划。面对新形势、新机遇、新挑战，我们必须增强又好又快发展的紧迫感、责任感和历史使命感，按照市委的部署全面推进政府各项工作，为实现发展目标，并为“十二五”发展开好头起好步而努力。

　　建议新一届市政府，坚持以邓小平理论和“三个代表”重要思想为指导，以科学发展观统领经济社会发展全局，坚决贯彻党中央、国务院和省委、省政府的决策部署以及市第十一次党代会精神，深入实施工业强市、民资外资富市、科教立市、环境优市、和谐兴市发展战略，大力推进“两滨驱动”、“一带联动”，深化改革开放，加快转变经济增长方式，加快现代化大城市建设步伐，加快社会各项事业发展，加快提高人民生活水平，加强精神文明和民主法制建设，努力建设工业名城、文化名市、生态侨乡、和谐江门，率先基本实现社会主义现代化。

　　今后五年我市经济社会发展的主要奋斗目标是：

　　——经济总量上再造一个江门。ＧＤＰ力争年均增长１４％左右。到２０１１年，ＧＤＰ、人均ＧＤＰ、地方财政一般预算收入比２００６年翻一番，分别达到１８００亿元、６０００美元、１００亿元。三次产业比例演进为６：５３：４１。

　　——现代化大城市建设实现重大进展。滨江新区布局框架初步形成，中心城区“北扩、南连、中优”建设取得突破性成果。形成铁路、轻轨、公路、水路、航空配套的立体交通网络，到２０１１年，全市公路和高速公路通车里程分别超９５００公里和４００公里，每百平方公里公路密度９９．５公里以上，形成全市１．５小时生活圈，新增铁路８６公里。全市内河干线航道完成升级整治，三级以上航道超过１８９公里。中心城市具备２个一级客运站和１个货运站，县级市具备１个二级以上客运站和１个等级货运站。

　　——工业名城建设实现重大进展。自主创新工作上新台阶，到２０１１年，全市高新技术产品增加值占工业增加值比重达３７％以上；建成产学研联合基地２２０个以上，各类科技孵化器１０家，引进科技成果转化项目１２００项以上。全市实现工业总产值４２００亿元。电子信息、机电、纺织服装、造纸、食品、建材六大支柱产业在全省占有重要地位，精密机械、汽车零配件、新材料、新能源、生物制药等先进制造业和临港重化工业成为新的产业增长点。打造出摩托车及汽车零配件、纺织化纤、五金制品、造纸等一批工业总产值超４００亿元的产业集群，银洲湖区域、江沙工业走廊、高新区、广海湾区域等一批工业总产值超３００亿元的区域，以及一批工业总产值超２００亿元的工业强镇和年产值超１００亿元的企业群，新增１０个以上中国名牌产品或驰名商标。成功创建“国家信息化示范城市”。

　　——内外源型经济发展实现重大进展。形成外源型经济和内源型经济相互促进、协调发展的新格局。民营经济发展环境全面优化，２０１１年民营工业总产值达到１９００亿元以上，占全市工业比重超过４５％。实施“十百千”工程，形成年产值超２０亿元的民营企业１０家以上、超２亿元的１００家以上、超２０００万元的１０００家以上。引进外资的规模和质量有较大提高，五年实际吸收外商直接投资４０亿美元以上。出口商品结构进一步优化，２０１１年机电产品和高新技术产品出口比重分别达６５％和１５％以上。

　　——社会主义建设实现重大进展。到２０１１年，全市８０％以上的镇（街）财政一般预算收入超千万元，超亿元镇（街）达１０个，城市与乡镇、边远后进地区与富裕地区协调发展。农民专业合作经济组织达２００家以上，农业增加值突破１００亿元，农业综合效益显著提高。建立城乡统一的户籍管理制度。镇到１０００人以上自然村实现通四级标准以上水泥路，镇、村自来水普及率达１００％，民用燃气普及率９０％。镇区生活垃圾和一般工业固体废物达标处置率分别达５０％和６０％，生活污水集中处理率达５０％。农村文化设施进一步完善，农民受教育程度和文化生活水平有较大提高，农村居民人均纯收入年均增长５％以上。

　　——文化名市和教育强市建设实现重大进展。全市文化综合实力和文化发展主要指标居全省前列。打响开平与村落、华人嘉年华等一批在国内外有较大影响力的侨乡文化品牌，创建３个以上“中国民间艺术之乡”。建成教育强市。２０１１年高中阶段教育毛入学率和高等教育毛入学率分别达８６％和３６％以上，２５周岁以上人口平均受教育年限达到１０年。城乡教育差距显著缩小，初步构建起全民学习、终身学习的学习型社会。各类人才净增１０万人，总量超过４０万人，人才综合竞争力高于全省平均水平。

　　——生态侨乡建设实现重大进展。建立起生态型城市基本框架。资源利用效率显著提高，单位ＧＤＰ能耗比２００６年降低１８％以上。主要污染物排放得到有效控制，化学需氧量和二氧化硫排放总量控制在省下达的指标内。林业生态县（区）达５个，森林覆盖率４４％以上。水环境质量显著改善，城镇供水和农村居民饮水安全保障体系完善。城镇环境基础设施进一步加强，县级以上城市和６０％的中心镇建成污水处理厂，城市生活垃圾无害化处理率和城镇生活污水处理率分别达８０％和７０％以上，城市绿化覆盖率和建成区绿化覆盖率分别达４５％和４０％，成为珠三角最佳人居城市之一。

　　——和谐江门建设实现重大进展。政府公共服务和社会管理水平有较大提高，民主法制建设和依法治市全面加强。公民思想道德、科学文化和健康素质明显提高，９０％以上社区达到平安和谐社区标准。市民的幸福感与满意度较高，创建国家文明城市。就业形势稳定，城镇登记失业率控制在３．８％以内。社会保障和救助体系、卫生服务体系、治安防控体系完善，社会治安保持平稳，安全生产完成省下达的指标任务，食品药品安全监管体系健全。国防动员机制进一步完善，民兵预备役建设和双拥工作巩固发展。人民生活水平不断提高，总体进入宽裕型小康阶段，城镇居民人均可支配收入年均增长７％。

　　按照上述总体要求，实现今后五年奋斗目标，必须做到“六个大力推进”，实现“六个全面加快”：

　　一是大力推进“两滨驱动”、“一带联动”，全面加快现代化大城市建设步伐。力争未来五年全市固定资产投入２０００亿元以上。高起点规划建设滨江新区，抓好１０平方公里启动区建设。破解中心城市交通瓶颈，建成滨江大道、胜利大桥、东海路贯通新会大道、西环路隧道等工程，规划建设市区环城快速干道、江顺大桥和连海大桥，形成市区快速交通网络。完善旧城区基础设施，促进江海区、新会区与中心城区的有机融合。大力发展滨江经济、临港经济、海洋经济，努力把滨江新区板块建成现代化大城市的新核心区，高新区和南新区板块建成高新技术产业和先进制造业的示范区，银洲湖板块建成国内外著名的纸业、石化和物流基地，广海湾板块建成以能源和重化工业为重点、以深水港为依托的新经济区；在３２５国道产业带，大力推进电子信息、摩托车及汽车零配件、纺织服装、五金卫浴、电声器材、印刷等特色产业集群建设，大力发展滨海、温泉与村落等旅游业，带动全市跨越式发展。

　　二是大力推进招商选资，全面加快内外源经济发展。充分利用我市的有利条件，特别是直购电试点政策，把招商引资提高到一个新水平。把好土地亩产效益和环保效益关，积极引进国内外大型企业集团特别是世界５００强企业，着力发展技术含量高、附加值高的高新技术和新兴产业项目，以及集聚配套、延接产业链的项目。加快服务业招商步伐，承接发达国家服务业转移。转变外贸增长方式，加快加工贸易转型升级，鼓励有条件的企业走出去，拓展国际市场。落实ＣＥＰＡ，推进与港澳在物流、旅游、金融、零售业和创意产业等领域的合作。积极参与泛珠三角区域合作。坚持放宽、搞活方针，消除民营经济发展的体制性障碍，支持民营企业做大做强。

　　三是大力推进自主创新和节能降耗，全面加快产业结构优化升级和经济增长方式转变。坚持引进消化吸收再创新，建立以企业为主体、市场为导向、产学研相结合的自主创新体系，鼓励企业加大创新投入，支持大中型企业建立技术研发中心，为中小企业提供技术创新公共平台服务。建立和完善科技人才的激励、培训和流动机制，鼓励创新人才向创新型企业集聚。加速人才引进和培养，扩大人才总量；提高高级人才和实用技术型人才的比重，优化人才结构；营造有利于优秀人才成长和干事创业的良好氛围。加强知识产权保护，为自主创新营造良好环境。坚持走可持续发展道路，完善节约资源、降耗增效的导向性政策，加快建立节约型、清洁型产业结构，创建国家循环经济示范城市。

　　四是大力推进社会主义建设，全面加快农业农村经济发展。落实工业反哺农业、城市支持农村和“多予、少取、放活”的方针，以农民增收、农村基础设施建设、农村文化、农村社会保障和固本强基“五大工程”为抓手，推动城市基础设施向农村延伸，城市公共服务向农村覆盖，城市文明向农村辐射。以加快中心镇、专业镇的工业化和城镇化进程为切入点，促进农村劳动力向二、三产业转移；以推进农业龙头企业和农民专业合作经济组织发展为切入点，提高农业综合效益和农民收入；以完善农村教育、卫生和文化等公共服务体系为切入点，改善农村生活环境，提高农村人口素质；以创建文明村镇为切入点，搞好村庄规划，推进村容村貌整治。加大扶贫力度，在财政转移支付和项目建设上给予农村落后地区更多支持。建立覆盖农村的社会保障和新型合作医疗网络，抓好农村卫生院和卫生站建设，建立无地、少地、失地农民基本养老、医疗保障制度，使广大农民病有所医、老有所养、安居乐业。

　　五是大力推进侨乡文化繁荣进步，全面加快文化名市建设。积极创建国家文明城市，深入开展社会主义荣辱观教育活动和以“爱国、守法、诚信、知礼”为主题的现代公民教育活动，弘扬“开拓创新、文明开放、勤劳创业、团结奉献”的侨乡精神。加强大学生思想政治教育和未成年人思想道德建设。倡导社会基本道德规范、科学精神和文明健康的生活方式。加强社会信用体系建设，构建诚信江门。统筹城乡、区域教育，努力建设教育强市。大力繁荣文化事业，推进文艺精品创作，加强对侨乡历史文化的发掘、研究、保护与开发，发展和推广五邑民间民俗文化。加快发展印刷、传媒、动漫等文化产业。

　　六是大力推进和谐江门建设，全面加快社会大局由基本稳定向平安和谐提升。坚持以人为本、民生优先，尽最大努力为群众办好事、做实事、解难事。深化分配制度改革，着力提高低收入者的收入水平，扩大中等收入者的比重，确保企业职工收入随企业经济效益提高而提高。建立健全城乡统一的社会保障体系。基本解决农村“五难”问题，完善各级财政对贫困家庭子女上学的资助体系。实施智力扶贫工程、农村青年技能培训工程、农村劳动力转移就业培训和退役士兵免费职业技能培训工程，启动全民科技素质行动计划，促进就业和再就业。完善城乡居民最低生活保障制度和对下岗职工、孤寡老人、残疾人及因灾因病返贫群体等的社会救助制度。整合城乡医疗卫生资源，健全多层次的医疗保障体系，切实解决看病难、看病贵问题。健全劳动关系调整机制，维护劳动者合法权益。创新社会管理体制，整合社会管理资源，提高社会管理水平。加强信访工作，健全矛盾纠纷排查调处机制。抓好平安社区建设，完善社会治安防控和公共安全防控体系。全面落实维稳工作责任制，建立健全公共安全预警和应急、安全生产以及保障食品药品安全的长效机制，筑牢和谐社会建设基础。

　　各位代表，展望我市未来五年的发展，前景更加美好。我们坚信，只要全市人民万众一心，共同努力，一定能把江门建设成为经济繁荣、生活富裕、社会和谐、环境优美的现代化大城市！

２００７年工作建议

　　今年是新一届政府的开局之年，也是发展的重要一年。全市经济社会发展的主要预期目标是：ＧＤＰ增长１４％，单位ＧＤＰ能耗比上年下降３％，地方财政一般预算收入增长１３％，全社会固定资产投资增长１５％，社会消费品零售总额增长１２．５％，外贸进出口增长１１％以上，居民消费价格总水平涨幅控制在３％左右，城镇登记失业率控制在３．５％以内，城镇居民人均可支配收入增长７％，农村居民人均纯收入增长５％。

　　为实现上述目标，建议新一届政府重点抓好以下工作：

　　一、提高科学发展水平，推动经济又好又快发展

　　（一）大力抓好“四大板块”和“一条经济带”建设，培育更多更强的经济增长点。

　　大力推进滨江新区建设。在确保滨江大道５月全线通车的同时，按照现代化城市综合功能的要求，抓好１０６平方公里范围的规划编制、论证和实施工作。抓好１０平方公里启动区规划建设的前期工作，落实土地调整，以市场化运作、争取上级补助等方式筹集建设资金。抓好江沙工业走廊的交通、水电、污水处理等配套设施建设，加快汽车零部件产业基地、中烟摩迪、鸿兴印刷等重大项目建设，做大做强摩托车及零配件、汽车零配件、五金卫浴、玻璃灯饰、钻石珠宝和印刷等产业。

　　大力开发广海湾。用足用好直购电试点政策，积极争取南方电网和省有关部门的支持，将直购电政策覆盖整个广海湾区域。高水平规划广海湾开发，以深水港、鱼塘港、台山电厂、台山核电站、再生资源工业集中区、新宁工业园为重点，以发展石化工业、装备制造业、物流业为主线，搞好功能布局。理顺管理体制，出台招商政策，充实招商队伍，加大招商力度，以招商促开发。完善以铁路、高速公路为重点的交通规划。加快推进台山电厂二期、台山核电站和川岛风电场的前期工作，壮大能源基地规模。

　　大力加快银洲湖建设。制定银洲湖区域控制性详细规划和岸线码头、交通网络、石化基地、物流基地、纸业基地、造船基地等专项规划。加强对大项目的招引力度，提升银洲湖的产业地位。促进海螺水泥、南洋船舶、李锦记、维达纸业、中顺纸业、华泰纸业等重点企业加快发展，加快新会ＬＮＧ电厂和古兜抽水蓄能电站等工程前期工作。

　　大力提升市高新区发展水平。以创建国家级高新区为契机，创新管理体制。加大载体建设力度，完善园区配套功能。重点发展电子信息、机电一体化、生化制药、新材料、精细化工等产业，延伸产业链，形成产业集群优势和规模优势。加快高新区科技孵化中心建设，努力把高新区建成珠三角高新科技产业的重点区域、科技创新中心和科技成果产业示范区。

　　大力发展３２５国道经济带产业集群。以电子信息、汽车零配件、精细化工、精密制造、液晶平板显示器配套、半导体光电、新型电池等先进制造业为发展重点，提升纺织、金属制品、食品、家电、建材、制鞋等产业的技术水平和产品档次。推进产业集群升级示范区建设，做大做强新会五金不锈钢制品、开平纺织服装和水暖卫浴、鹤山印刷和新材料、恩平麦克风及电声器材等国家级产业基地。

　　（二）以提高自主创新能力和节约资源能源为重点，加快转变经济增长方式。

　　推进自主创新体系建设。加大自主创新投入，全市财政安排科技投入１．６８亿元，比上年增长１２％，其中市本级财政投入增长１８．０８％。组织企业实施２００项市级科技计划项目，重点扶持５０家大中型企业，培育２０家高新技术企业，推动１５家传统企业向高新技术企业转型。打造产业服务平台，重点推进摩托车、电子信息、麦克风、不锈钢、五金卫浴、纺织服装等行业的技术创新、质量技术标准和检测检验、信息、融资以及专业市场展销等服务平台建设。加快广天机电工业研究院和华南精细化工研究院永久院区建设，力争建成国家摩托车检测中心。坚持企业技术改造联席会议制度，开展重点产业技术升级行动，降低技术改造审核门槛，简化审核程序，落实技术改造税收优惠政策，鼓励企业加大技改投入。拓展技改项目银企信贷合作平台和信息沟通平台，推动银企开展技改项目信贷合作。

　　积极实施品牌带动战略。大力培育发展自主知识产权和自有品牌，鼓励企业争创国家和省名牌产品及商标，打造区域产业品牌。加强商标权、专利权等知识产权和名牌产品的保护，推广国际标准，鼓励优势企业积极参与行业、国家和国际标准的制订。

　　大力发展节约型产业。推进以节能、节地、节材、节水为重点的经济与资源协同发展战略，强力实施重点企业节能行动。扎实推进省级循环经济试点城市建设，制定再生资源回收利用管理办法，推广乳化焦浆等新型清洁燃料，做大循环经济规模，抓好双水电厂、国洋电厂热电联供及双水拆船、甘化股份国家级循环经济试点，争取银洲湖纸业基地列入国家循环经济示范项目。加快建筑墙体材料革新，推动新建住宅和公共建筑节能。

　　加快发展以物流、金融、旅游、动漫为重点的现代服务业，提高城市经济发展质量。落实物流业发展规划，突出抓好银洲湖、广海湾、鹤山桃源等现代物流基地建设，办好保税物流中心，推动第三方物流加快发展。重点抓好新会不锈钢、鹤山鞋业鞋材、开平水暖卫浴和纺织服装、恩平麦克风等专业市场的规划建设。整合旅游资源，加快潮连钻石岛、恩平良西生态园、开平赤水温泉、银湖湾湿地公园等新旅游景点的开发，发展生态游、休闲游等特色旅游，办好侨乡旅游节，打响温泉海岛、山湖、人文等旅游品牌。加强银政、银企合作，改善金融环境，完善中小企业信用数据库、企业信息征信系统和个人信用采集基础数据库；鼓励外资、民资金融机构来我市发展业务和开设分支机构，促进中小企业信用担保机构发展，扶持有条件的企业利用资本市场筹集资金。大力发展连锁经营、电子商务等新型营销方式，加快传统服务业改造升级。积极引进国内外企业集团的营运中心、研发中心、服务中心，发展总部经济。培育有利于文化创意产业发展的环境，重点扶持影视动漫创意群体，引进知名动漫企业，加快动漫产业基地建设，做长影视动漫产业链。加强对房地产市场的正确引导和规范管理，促进建筑业和房地产业健康有序发展。

　　（三）以发展农村经济为重点，全面推进社会主义

　　加大“三农”工作力度，确保各项支农惠农政策的落实。今年全市各级财政预算用于“三农”的支出为１１．９３亿元，比上年增加１．４２亿元，增长１３．５０％，占总预算支出１９．４６％，其中市本级财政投入增长３２．９９％。凡财政安排给农民的补助补偿资金，由各市、区财政部门委托金融机构或镇（街）财政所直接付给符合条件的对象。积极拓展农民收入持续较快增长的途径。大力推进“异地发展”模式，积极开展经济发展“结对子”活动，推动薄弱镇村经济加快发展，年内增加财政收入超千万元镇３－５个，集体年收入３万元以下的贫困村全部脱贫。

　　加快农村工业化、城镇化步伐。大力发展镇村经济，促进中心镇和专业镇向卫星城和工业强镇转变，推进农村市场体系建设，提高小城镇吸纳农村富余劳动力的承载能力，引导农村富余劳动力向二、三产业转移，建立镇级农村富余劳动力转移就业培训定点机构１６个以上，培训农村富余劳动力３．３８万人，培训就业率要达７０％以上，培养和造就“有文化、守法纪、懂技术、会经营”的新型农民。

　　推动传统农业向现代农业转变。加快推进农业产业化，发展３－５家市级以上农业龙头企业和２０个农民专业合作组织。推广农业新品种、新技术，提高农业生产机械化水平和农产品良种覆盖率。积极发展特色农业、品牌农业和高效农业。加强农产品质量体系和标准化建设。整治改造中低产田５．７４万亩。

　　提高农村规划建设水平。抓好社会主义示范村建设。按照规划市管到镇、县管到村的要求，制定既先进又符合实际的<建设规划方案。以创建生态文明村为载体，突出抓好改水、改厕、改路、改灶、改房和农村生活垃圾处理等工作，推广沼气应用。加快农村信息化进程。切实推进以城乡水利防灾减灾和省人大水利议案工程为重点的水利建设，提高防灾抗灾能力。抓好乡镇综合配套改革试点，积极化解镇村历史债务。

　　（四）突出抓好招商选资和基础设施建设，增强经济发展后劲。

　　加大招商选资力度。把“亩产效益”、“环保效果”、“链条效应”作为提高招商引资质量的关键环节来抓。立足港澳台，面向日韩，开拓欧美，打响直购电招商牌，抓好产业招商，力争全年实际利用外商直接投资超过７亿美元。创新招商引资方式，拓展与世界银行、香港贸发局、外国使领馆、华侨社团、重点招商地区商会和行业协会的联系，拓宽以侨引商、以商引商、中介引商渠道，在珠三角发达地区设立招商点，进行驻地招商。根据产业特点设立专业招商小组，提高专业招商水平。整合招商引资信息资源，建立全市招商信息共享平台。扩大对外贸易，进一步优化出口产品结构，积极开拓多元化国际市场。落实民营经济扶持政策。鼓励民营企业参与国有企业并购重组和进入城市公用事业、基础设施、金融服务等领域。扶持２家以上企业在境内外证券市场上市，支持春晖股份公司重组，提高上市公司竞争力。完善对国有资产经营者的激励机制和约束机制，国有资产收益比上年提高１０％以上。

　　加强城乡基础设施建设。启动江门市区环城快速干道规划建设，加快东海路贯通新会大道的建设和江顺大桥、西环路隧道的前期工作。配合完成江珠高速公路工程，推进江肇高速公路和佛开高速公路扩建工程，完成江新、新开高速公路可行性研究工作，争取尽早批准立项。配合抓好广珠城际轨道交通工程江门支线和广珠铁路江门段建设，推进广东西部沿海铁路建设的前期工作。加快劳龙虎水道和崖门水道５０００吨级航道整治工程。

　　二、繁荣发展社会事业，推进文化名市建设

　　加快发展文化事业。以创建国家文明城市为抓手，深入开展群众性精神文明创建活动。加大公益性文化事业投入，抓好江门电视中心、市新青少年宫、鹤山公园、台山塔山公园等文化基础设施建设。大力开展文化下乡、文化进社区活动，继续抓好广场文艺演出活动，办好五邑华侨华人博物馆和江门美术馆馆展。加强对侨乡文化的研究与宣传，创作体现五邑民间艺术特色的节目，推出一批能获国家级奖和省级奖的文学艺术精品。继续推进开平与村落申报世界文化遗产的后续保护开发工作，确保今年成功列入世界文化遗产名录。抓好江门长堤历史街区、江门新宁铁路遗址、台山洋楼、开平赤坎旧镇等华侨建筑群的调查、挖掘、整理和申报升级，开展非物质文化遗产普查。

　　抓好教育和人才工作。全市各级财政预算的教育投入１３．２１亿元，比上年增长１２％，其中市本级财政投入增长２４．６６％。推进城乡教育均衡发展，加大城乡教师双向交流的力度。巩固和提高九年义务教育和高中阶段教育。大力发展民办教育和职业教育。提高五邑大学、江门职业技术学院和江门电视大学办学质量。优化人才结构，积极发展境外培训，促进人才培养国际化。发挥老科技工作者和中青年专家的作用。开展老年人继续教育工作，办好老干部大学和老年人大学。

　　推进体育、人口计生等工作。切实保障妇女、未成年人合法权益。大力发展体育事业和体育产业，广泛开展全民健身活动。完善人口与计划生育目标管理责任制，稳定低生育水平，提高出生人口素质，综合治理出生人口性别比偏高问题，人口自然增长率控制在５．８‰以内。巩固双拥优抚安置工作成果，争创“全国双拥模范城”四连冠。提高殡葬管理水平。做好新形势下侨务、外事、港澳台事务工作，推进新闻、出版、人防、气象、宗教、老龄、档案、方志等工作。

　　三、坚持发展为民，努力建设和谐江门

　　把民生问题作为政府工作首要解决的问题，继续深入实施“十项民心工程”，切实增加财政资金对公共教育、公共医疗卫生、公共救济、公共交通、公共安全等方面的投入，保障基本公共服务的需要。

　　（一）加大民生投入，扩大改革发展成果惠及面。

　　今年各级财政对改善民生的投入总额１３．１２亿元，比上年增加３．２１亿元，增长３２．３９％。

　　着力解决就业和再就业问题。全市各级财政投入２５００万元，新增就业岗位２万个，安置城镇劳动力就业和下岗失业人员再就业２．５万人。进一步完善和落实扶持就业再就业政策，建立城乡统一的公共就业服务体系，健全对困难群众的就业援助制度，对所有下岗失业人员予以免费培训，对农村年人均收入１５００元以下的贫困户劳动力进行免费培训并推荐就业，年内建立再就业定点培训机构２５家以上。加强高校毕业生就业指导服务，拓宽毕业生就业领域。投入４９０万元对城镇退役士兵进行免费技能培训。建立社区弹性就业服务平台，积极推行非全日制工、季节工、短期工等灵活就业形式。推进劳动力市场与人才市场的资源整合，健全劳动力市场信息网络，办好劳务集市。按照国家工资改革政策，适当提高教师和公务员待遇。

　　着力加强公共卫生体系建设。全市各级财政公共卫生投入３．１５亿元，比上年增加３４００万元，其中市本级财政投入增长２６．２８％。市本级财政投入１０００万元引导资金推动人大“农村合作医疗”议案的落实；投入３５０万元用于结核病、皮肤病、职业病防治和疫苗接种等公共卫生事业；投入２８０万元对弱势群体实施医疗救助；新增投入１５０万元推进中医药名市建设；新增投入３１２万元推进农村卫生站和社区卫生服务机构建设。健全疾病预防控制体系和卫生监督体系，严密防控重大传染病。切实提高参合农民的保障水平，积极推行即时报销和双向转诊制度，全市人均筹资标准达到６０元以上，住院报销封顶线达到６０００元以上。进一步扩大市农村合作医疗特别救助基金的救助范围。实行农村合作医疗中医药门诊优惠。推进“每村一个卫生站、一名医生、年补贴一万元”建设计划。鼓励有行医资格的人员开办私人诊所。

　　着力落实农村全免费义务教育政策。今年全市各级投入２．０２亿元用于农村免费义务教育，比上年增长１．５倍，在免收农村义务教育阶段学杂费的基础上免收学生书本费，把渔民户口纳入全免费义务教育范畴。

　　着力抓好“畅通工程”建设。全市各级计划投入５４８６万元，改造农村公路１３０公里，建成１５４８个农村客运站点，１００％行政村通班车。制订并实施公交优先的公共交通发展规划，增加公交线路和班次，辟建公交专用道，多建公共停车场。完成江门、新会新车站一体化枢纽站建设。完善江门市区交通设施，完成江珠高速至东海路段双向四车道、桐乐路二期、新港大道二期、新会东庆南路延伸线工程，启动胜利大桥工程，推进中区大道建设。

　　着力营造良好的生活环境。认真实施生态市建设规划，积极开展生态示范村、全国环境优美乡镇、林业生态县、绿色小康县、全国绿化模范城市创建及红树林省级自然保护区的建设和申报工作。加强对银洲湖湿地生态，白水带、大西坑、圭峰山、古兜山、北峰山、大雁山、七星坑等森林生态的保护。加快建设文昌沙污水处理厂二期、新会区东郊污水处理厂二期工程和江海、潮连、棠下、司前污水处理厂及丰乐、双水镇和广海镇污水处理厂的集污管网，年内建成台山、开平、鹤山（二期）和恩平的城市污水处理厂及荷塘镇、共和镇、水口镇的生活污水处理厂。抓好市区新垃圾处理场建设。严格执行排污许可证和污染物总量控制制度，禁止无证或超量排污。做好燃煤燃油废气污染防治。抓好对饮用水源的保护，加强对西江、潭江等重点河段、天沙河、会城河、沙坪河、通济河等重要河涌以及新会、台山近岸海域环境、海洋生态的综合整治和保护。发动社会力量参与环境保护的监督，对工业企业环境违法行为实行举报有奖制度，定期公布辖区和主要行业的能源消耗、污染排放情况，做好国家环保模范城市复检工作。

　　着力解决困难群众生活问题。全市各级财政投入低保资金４２００万元，比上年增加４５０万元，提高低保补助标准，实现应保尽保。提高五保人员的生活保障水平。做好２００７年慈善公益万人行组织工作。关心残疾人等困难群体。建立被征地农民基本养老、医疗保障制度。落实社保扩面征缴联动执法机制，依法管好用好社会保险基金。扩大住房补贴和住房公积金归集面，增加廉租房建设，年内全面解决全市城镇人均居住面积８平方米以下困难户的住房问题和全市农村居民住房难的问题。落实市直困难企业退休干部生活费、离退休劳模津贴及早期离开国有集体企业人员的社会养老保险。市本级财政投入２７０万元，补贴市直困难企业军转干部，投入５４４万元解决老年人乘车、孤残儿就医就读及单亲家庭危房救助等问题。建立健全贫困学生助学体系，保证每个家庭的子女都不因贫失学。继续做好水库移民后期扶持工作。

　　（二）提高社会管理水平，推进平安江门建设。

　　加强城市管理。坚持“建管并举、重在管理”，完善“两级政府、三级管理、四级网络”管理体制和“综合执法、部门协作、市区联动、依法管理、堵疏结合、全面覆盖”的城市长效管理体系，推广蓬江区“数字城管”经验，完善城市管理工作重心向街道、社区下移工作。重点解决好市民投诉较多的油烟噪声污染、占道经营、违法建筑、社会治安等问题。加快市政公用事业市场化步伐。

　　健全人民内部矛盾化解机制。认真贯彻《信访条例》，落实领导包案制度，坚持信访联席会议制度，完善市直部门领导公开接访制度，公开接访时间统一为每月１５日。加快信访信息系统建设。健全调解、接访、听证、仲裁机制，重点解决企业转制、土地征用、城镇拆迁等引发的突出问题。引导群众按法律程序合理表达利益诉求，从源头上预防和减少群体性事件的发生。

　　加大平安和谐社区建设力度。把社区公共设施和公共服务的投入纳入各级财政预算，力争４０％以上社区达到平安和谐社区标准。建立健全小区物业管理与业主自治相结合的管理模式。加快推进社区矫正工作。按照“村不漏户、户不漏房、房不漏人”的要求，加强出租屋和流动人口管理。加强对新兴的社会组织、民间组织及文化娱乐、网吧、废旧物品收购等行业的管理。加大社会治安视频监控系统和“金盾”工程建设力度，逐步实现视频监控网络覆盖到全市所有主要街道和治安重点场所。发挥保安队、治安队、基层治保会等群防群治力量的协防作用。开展对校园、大型企业和金融营业网点周边及暂住人口聚居区等重点地区的专项治安整治。坚决打击黑恶势力和“两抢一盗”、“黄赌毒”、非法传销等违法犯罪活动。

　　建立健全公共安全管理机制。严格落实安全生产责任制和安全生产许可制度，健全企业注册安全主任制度和岗前培训制度，完善安全隐患举报和重大安全隐患公示、督办、整改责任制度，抓好镇（街）安监专业队伍建设，加强重大危险源监控和重大隐患排查整治。提高食品药品监督管理水平，严厉打击制售假冒伪劣商品行为，规范市场秩序。建设市应急信息平台，健全公共安全预警机制、应急机制和救援体系，提高防范和处置突发事件的能力。

　　四、加强政府自身建设，努力建设人民满意政府

　　建设创新型政府。以提高公共服务和社会管理水平作为政府工作创新的切入点，重点解决公务员队伍中存在的办事拖拉、推诿、工作质量不高、服务不到位等问题，在市直机关全面导入ＩＳＯ质量管理体系，提高ＩＳＯ质量管理体系运行监管水平。

　　建设法治型政府。认真听取人大代表、政协委员、各民主党派、人民团体、无党派人士的建议和意见，畅通人民群众发表意见、反映问题的言路。健全重大决策和重大项目规划的论证和听证程序。坚持经济形势分析联席会议和经济社会发展顾问咨询制度，提高科学民主决策水平。提高政府全体工作人员的法律意识和依法行政能力，规范行政执法行为。进一步深化行政审批制度改革。建立和完善财政监督体系，切实依法理财。配合人大加强对部门预算在线监督工作，建立对基建、社保、支农等重点专项资金事前监控、事后检查与评价相结合的监督链条。完善乡镇激励型财政管理体制、部门预算编制工作制度和国库集中支付制度，扩大财政资金使用绩效评价范围。

　　建设服务型政府。努力解决市民关注的热点难点问题，把“１２３４５”政府服务热线覆盖面扩大到各县级市（区）。加大督查工作力度，形成用制度规范行政行为、推进工作落实的长效机制。加强部门协作配合，落实“首问负责制”和推诿、搁置等行政过错责任追究制。加快电子政务建设，推进办公自动化和公共服务信息化。加强公务员队伍建设，提高公务员队伍的综合素质和为民服务水平，营造团结和谐的干事氛围。

　　建设廉洁型政府。落实廉政责任制，规范各级领导干部从政行为。坚决整治损害群众利益的不正之风，依法治理商业贿赂。严格执行建设工程项目招投标、土地使用权出让、产权交易、政府采购等制度，从源头上防止腐败。

　　各位代表！回顾过去，我们倍感自豪；展望未来，我们满怀信心。让我们在省委、省政府和江门市委的正确领导下，从新的历史起点出发，齐心协力，乘势而上，开拓创新，扎实工作，努力实现侨乡经济社会又好又快发展，向全市人民交出一份更加满意的答卷！
